# Aquachloridobis[2-(1,3-thia­zol-4-yl-κ*N*)-1*H*-benzimidazole-κ*N* ^3^]nickel(II) nitrate

**DOI:** 10.1107/S1600536812029728

**Published:** 2012-07-04

**Authors:** Peng Liang, Wei Wu, Wei-Man Tian, Xian-Hong Yin

**Affiliations:** a Department of Chemistry and Chemical Engineering, Guangxi University for Nationalities, Nanning 530006, People’s Republic of China

## Abstract

In the title compound, [NiCl(C_10_H_7_N_3_S)_2_(H_2_O)]NO_3_, the Ni^II^ ion is coordinated by four N atoms from two chelating 2-(1,3-thia­zol-4-yl)-1*H*-benzimidazole ligands, one Cl atom and one water mol­ecule in a distorted octa­hedral geometry. In the crystal, O—H⋯O, N—H⋯O and N—H⋯Cl hydrogen bonds link the complex cations and nitrate anions into a three-dimensional network. π–π inter­actions between the thia­zole and imidazole rings and between the thia­zole and benzene rings are observed [centroid–centroid distances = 3.592 (3) and 3.735 (3) Å].

## Related literature

For background to the synthesis and properties of benzimidazole derivatives, see: Agh-Atabay *et al.* (2003 ▶); Devereux *et al.* (2004 ▶); Inoue *et al.* (2002 ▶). For a related structure, see: Mothilal *et al.* (2004 ▶).
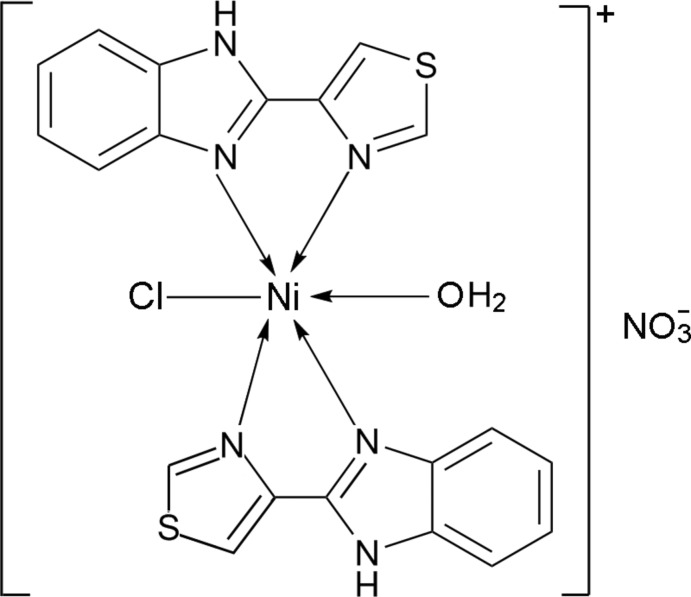


## Experimental

### 

#### Crystal data

[NiCl(C_10_H_7_N_3_S)_2_(H_2_O)]NO_3_
*M* *_r_* = 576.68
Monoclinic, 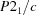

*a* = 16.091 (5) Å
*b* = 11.189 (3) Å
*c* = 13.931 (3) Å
β = 113.275 (3)°
*V* = 2304.1 (11) Å^3^
*Z* = 4
Mo *K*α radiation
μ = 1.18 mm^−1^
*T* = 296 K
0.26 × 0.25 × 0.24 mm

#### Data collection

Bruker SMART APEX CCD diffractometer
Absorption correction: multi-scan (*SADABS*; Sheldrick, 1996 ▶) *T*_min_ = 0.748, *T*_max_ = 0.764
11654 measured reflections
4050 independent reflections
3129 reflections with *I* > 2σ(*I*)
*R*_int_ = 0.072

#### Refinement

*R*[*F* ^2^ > 2σ(*F* ^2^)] = 0.054
*wR*(*F* ^2^) = 0.174
*S* = 1.07
4050 reflections
316 parameters
H-atom parameters constrained
Δρ_max_ = 0.80 e Å^−3^
Δρ_min_ = −0.53 e Å^−3^

### 

Data collection: *SMART* (Bruker, 2007 ▶); cell refinement: *SAINT* (Bruker, 2007 ▶); data reduction: *SAINT*; program(s) used to solve structure: *SHELXS97* (Sheldrick, 2008 ▶); program(s) used to refine structure: *SHELXL97* (Sheldrick, 2008 ▶); molecular graphics: *DIAMOND* (Brandenburg, 1999 ▶); software used to prepare material for publication: *SHELXTL* (Sheldrick, 2008 ▶).

## Supplementary Material

Crystal structure: contains datablock(s) I, global. DOI: 10.1107/S1600536812029728/hy2553sup1.cif

Structure factors: contains datablock(s) I. DOI: 10.1107/S1600536812029728/hy2553Isup2.hkl

Additional supplementary materials:  crystallographic information; 3D view; checkCIF report

## Figures and Tables

**Table 1:** Hydrogen-bond geometry (Å, °)

*D*—H⋯*A*	*D*—H	H⋯*A*	*D*⋯*A*	*D*—H⋯*A*
O1—H1*A*⋯O3^i^	0.84	2.00	2.751 (5)	149
O1—H1*B*⋯O2^ii^	0.79	2.53	3.293 (6)	160
O1—H1*B*⋯O4^ii^	0.79	2.38	3.025 (5)	139
N4—H4⋯O3^iii^	0.86	2.35	2.962 (5)	129
N4—H4⋯O4^iii^	0.86	2.14	2.996 (5)	173
N7—H7⋯Cl1^iv^	0.86	2.35	3.159 (4)	157

Symmetry codes: (i) 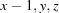; (ii) 
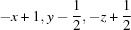
; (iii) 
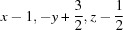
; (iv) 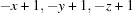.

**Fig. 1.**
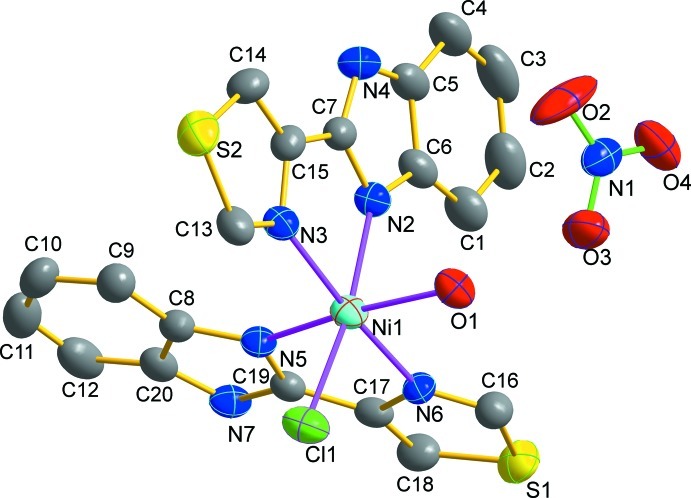
The Molecular structure of the title compound. Displacement ellipsoids are drawn at the 50% probability level. H atoms have been omitted for clarity.

**Fig. 2.**
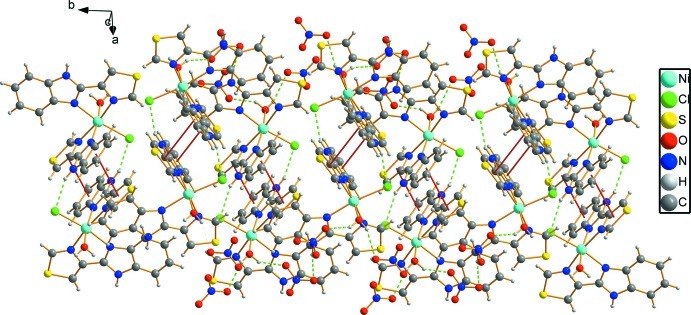
Crystal packing of the title compound. Dashed lines denote hydrogen bonds.

## supplementary crystallographic information

### Comment

Thiabendazole (TBZ) aroused considerable interest in biology and medicine due to its antiproliferative activities. It is an antimicrobial drug belonging to the benzimidazole derivatives and exhibits wide applications in human and veterinary medicine (Agh-Atabay *et al.*, 2003; Devereux *et al.*, 2004; Inoue *et al.*, 2002). As part of our studies of the synthesis and characterization of these compounds, we report here the synthesis and crystal structure of the title compound (Fig. 1).

In the crystal, intermolecular O—H···O, N—H···O and N—H···Cl hydrogen bonds link the complex cations and nitrate anions into a three-dimensional network (Table 1, Fig. 2) (Mothilal *et al.*, 2004). π–π interactions between the thiazole and imidazole rings and between the thiazole and benzene rings are observed [centroid–centroid distances = 3.592 (3) and 3.735 (3) Å]. In the complex cation, the two ligand planes are nearly perpendicular to each other, with a dihedral angle of 89.14 (7)°.

### Experimental

A solution of TBZ (0.201 g, 1 mmol) in 3 ml DMF was added dropwise with stirring at room temperature to a solution of Ni(NO_3_)_2_.6H_2_O (0.290 g, 1 mmol) and NiCl_2_.6H_2_O (0.237 g, 1 mmol) in a mixture of 10 ml water and 5 ml ethanol. Then an aqueous solution of sodium hydroxide was added dropwise with stirring to adjust the pH value of the solution being 6. The resulting mixture was sealed in a 23 ml Teflon-lined stainless reactor, kept under autogenous pressure at 403 K for 72 h, and then slowly cooled to room temperature at a rate of 5 K per hour. Dark-green block crystals suitable for X-ray diffraction were isolated, washed with ethanol and dried in air (yield: 65% based on Ni).

### Refinement

H atoms on C and N atoms were positioned geometrically and refined as riding atoms, with C—H = 0.93 and N—H = 0.86 Å and with *U*_iso_(H) = 1.2*U*_eq_(C, N). The water H atoms were located from a difference Fourier map and refined as riding with *U*_iso_(H) = 0.079 Å^2^.

### Crystal data

**Table:**

[NiCl(C_10_H_7_N_3_S)_2_(H_2_O)]NO_3_	*F*(000) = 1176
*M**_r_* = 576.68	*D*_x_ = 1.662 Mg m^−^^3^
Monoclinic, *P*2_1_/*c*	Mo *K*α radiation, λ = 0.71073 Å
Hall symbol: -P 2ybc	Cell parameters from 2584 reflections
*a* = 16.091 (5) Å	θ = 2.3–26.6°
*b* = 11.189 (3) Å	µ = 1.18 mm^−^^1^
*c* = 13.931 (3) Å	*T* = 296 K
β = 113.275 (3)°	Block, green
*V* = 2304.1 (11) Å^3^	0.26 × 0.25 × 0.24 mm
*Z* = 4	

### Data collection

**Table:**

Bruker SMART APEX CCD diffractometer	4050 independent reflections
Radiation source: fine-focus sealed tube	3129 reflections with *I* > 2σ(*I*)
Graphite monochromator	*R*_int_ = 0.072
φ and ω scans	θ_max_ = 25.0°, θ_min_ = 1.4°
Absorption correction: multi-scan (*SADABS*; Sheldrick, 1996)	*h* = −18→19
*T*_min_ = 0.748, *T*_max_ = 0.764	*k* = −13→13
11654 measured reflections	*l* = −14→16

### Refinement

**Table:**

Refinement on *F*^2^	Primary atom site location: structure-invariant direct methods
Least-squares matrix: full	Secondary atom site location: difference Fourier map
*R*[*F*^2^ > 2σ(*F*^2^)] = 0.054	Hydrogen site location: inferred from neighbouring sites
*wR*(*F*^2^) = 0.174	H-atom parameters constrained
*S* = 1.07	*w* = 1/[σ^2^(*F*_o_^2^) + (0.083*P*)^2^ + 1.4424*P*] where *P* = (*F*_o_^2^ + 2*F*_c_^2^)/3
4050 reflections	(Δ/σ)_max_ = 0.045
316 parameters	Δρ_max_ = 0.80 e Å^−^^3^
0 restraints	Δρ_min_ = −0.53 e Å^−^^3^

### Special details

**Table:**

Geometry. All e.s.d.'s (except the e.s.d. in the dihedral angle between two l.s. planes) are estimated using the full covariance matrix. The cell e.s.d.'s are taken into account individually in the estimation of e.s.d.'s in distances, angles and torsion angles; correlations between e.s.d.'s in cell parameters are only used when they are defined by crystal symmetry. An approximate (isotropic) treatment of cell e.s.d.'s is used for estimating e.s.d.'s involving l.s. planes.
Refinement. Refinement of *F*^2^ against ALL reflections. The weighted *R*-factor *wR* and goodness of fit *S* are based on *F*^2^, conventional *R*-factors *R* are based on *F*, with *F* set to zero for negative *F*^2^. The threshold expression of *F*^2^ > σ(*F*^2^) is used only for calculating *R*-factors(gt) *etc*. and is not relevant to the choice of reflections for refinement. *R*-factors based on *F*^2^ are statistically about twice as large as those based on *F*, and *R*- factors based on ALL data will be even larger.

### Fractional atomic coordinates and isotropic or equivalent isotropic displacement parameters (Å^2^)

**Table:**

	*x*	*y*	*z*	*U*_iso_*/*U*_eq_	
Ni1	0.26114 (3)	0.47368 (5)	0.32612 (4)	0.0352 (2)	
Cl1	0.31740 (8)	0.27662 (10)	0.40665 (10)	0.0490 (4)	
S1	0.36620 (9)	0.71229 (11)	0.62788 (10)	0.0507 (4)	
S2	0.13043 (9)	0.27528 (12)	0.00588 (11)	0.0552 (4)	
O1	0.13949 (19)	0.4517 (3)	0.3478 (3)	0.0412 (7)	
O3	1.0666 (2)	0.6635 (3)	0.3784 (3)	0.0606 (10)	
O4	1.0018 (2)	0.8339 (3)	0.3421 (3)	0.0693 (11)	
O2	0.9808 (3)	0.7045 (5)	0.2204 (3)	0.0964 (18)	
N3	0.2001 (2)	0.3971 (3)	0.1739 (3)	0.0344 (8)	
N5	0.3884 (2)	0.5240 (3)	0.3241 (3)	0.0313 (8)	
N6	0.3205 (2)	0.5777 (3)	0.4687 (3)	0.0324 (8)	
N2	0.2024 (2)	0.6272 (3)	0.2292 (3)	0.0343 (8)	
N7	0.5158 (2)	0.6289 (3)	0.3975 (3)	0.0370 (9)	
H7	0.5562	0.6742	0.4414	0.044*	
N4	0.1133 (2)	0.6929 (3)	0.0713 (3)	0.0430 (9)	
H4	0.0801	0.6916	0.0054	0.052*	
N1	1.0157 (2)	0.7334 (4)	0.3117 (3)	0.0447 (10)	
C8	0.4375 (3)	0.5129 (4)	0.2623 (3)	0.0343 (10)	
C15	0.1532 (3)	0.4763 (4)	0.0953 (4)	0.0368 (10)	
C20	0.5192 (3)	0.5792 (4)	0.3096 (4)	0.0371 (10)	
C7	0.1553 (3)	0.5995 (4)	0.1300 (3)	0.0339 (9)	
C19	0.4372 (2)	0.5941 (3)	0.4029 (3)	0.0321 (9)	
C17	0.4038 (2)	0.6273 (3)	0.4827 (3)	0.0327 (9)	
C6	0.1899 (3)	0.7514 (4)	0.2356 (4)	0.0388 (10)	
C13	0.1927 (3)	0.2884 (4)	0.1364 (4)	0.0419 (11)	
H13	0.2196	0.2229	0.1783	0.050*	
C5	0.1327 (3)	0.7920 (4)	0.1353 (4)	0.0441 (11)	
C18	0.4377 (3)	0.7027 (4)	0.5649 (4)	0.0421 (11)	
H18	0.4923	0.7435	0.5842	0.051*	
C11	0.5629 (4)	0.5228 (5)	0.1731 (5)	0.0571 (14)	
H11	0.6040	0.5248	0.1412	0.069*	
C1	0.2222 (3)	0.8290 (4)	0.3202 (4)	0.0495 (12)	
H1	0.2602	0.8024	0.3862	0.059*	
C12	0.5820 (3)	0.5849 (5)	0.2636 (5)	0.0540 (14)	
H12	0.6349	0.6294	0.2935	0.065*	
C14	0.1106 (3)	0.4261 (5)	−0.0009 (4)	0.0467 (12)	
H14	0.0759	0.4676	−0.0613	0.056*	
C10	0.4830 (4)	0.4559 (5)	0.1265 (4)	0.0507 (12)	
H10	0.4723	0.4144	0.0649	0.061*	
C9	0.4209 (3)	0.4511 (4)	0.1705 (4)	0.0443 (11)	
H9	0.3681	0.4069	0.1392	0.053*	
C16	0.2942 (3)	0.6140 (4)	0.5385 (4)	0.0412 (11)	
H16	0.2400	0.5892	0.5412	0.049*	
C4	0.1057 (4)	0.9106 (5)	0.1180 (5)	0.0562 (14)	
H4A	0.0679	0.9375	0.0520	0.067*	
C2	0.1958 (4)	0.9471 (4)	0.3023 (5)	0.0612 (15)	
H2	0.2169	1.0019	0.3569	0.073*	
C3	0.1367 (4)	0.9856 (4)	0.2012 (6)	0.0661 (18)	
H3	0.1186	1.0652	0.1916	0.079*	
H1A	0.1016	0.5069	0.3358	0.079*	
H1B	0.1000	0.4040	0.3241	0.079*	

### Atomic displacement parameters (Å^2^)

**Table:**

	*U*^11^	*U*^22^	*U*^33^	*U*^12^	*U*^13^	*U*^23^
Ni1	0.0293 (3)	0.0361 (3)	0.0344 (4)	−0.0022 (2)	0.0065 (3)	−0.0048 (2)
Cl1	0.0429 (6)	0.0411 (6)	0.0506 (8)	0.0038 (5)	0.0053 (5)	0.0024 (5)
S1	0.0636 (8)	0.0451 (6)	0.0402 (8)	−0.0015 (5)	0.0170 (6)	−0.0142 (5)
S2	0.0516 (7)	0.0592 (8)	0.0476 (8)	−0.0047 (6)	0.0119 (6)	−0.0238 (6)
O1	0.0344 (15)	0.0363 (15)	0.051 (2)	−0.0037 (12)	0.0148 (14)	−0.0003 (14)
O3	0.072 (2)	0.054 (2)	0.046 (2)	0.0172 (18)	0.0134 (19)	0.0004 (17)
O4	0.059 (2)	0.057 (2)	0.093 (3)	0.0218 (18)	0.032 (2)	0.011 (2)
O2	0.090 (3)	0.159 (5)	0.025 (2)	−0.052 (3)	0.006 (2)	−0.010 (2)
N3	0.0294 (16)	0.0339 (17)	0.034 (2)	−0.0017 (14)	0.0065 (15)	−0.0072 (15)
N5	0.0255 (16)	0.0344 (17)	0.029 (2)	−0.0029 (13)	0.0058 (15)	−0.0020 (15)
N6	0.0306 (17)	0.0380 (18)	0.025 (2)	0.0000 (14)	0.0077 (15)	−0.0059 (15)
N2	0.0346 (17)	0.0303 (17)	0.035 (2)	−0.0006 (14)	0.0104 (16)	−0.0017 (15)
N7	0.0291 (16)	0.0394 (18)	0.036 (2)	−0.0067 (14)	0.0055 (16)	0.0033 (16)
N4	0.042 (2)	0.045 (2)	0.036 (2)	0.0034 (17)	0.0087 (17)	0.0068 (18)
N1	0.0357 (19)	0.061 (3)	0.038 (2)	−0.0123 (18)	0.0146 (18)	0.001 (2)
C8	0.032 (2)	0.035 (2)	0.033 (2)	0.0016 (17)	0.0102 (19)	0.0018 (18)
C15	0.032 (2)	0.045 (2)	0.032 (3)	−0.0053 (17)	0.0113 (19)	−0.0058 (19)
C20	0.034 (2)	0.034 (2)	0.044 (3)	0.0039 (17)	0.0153 (19)	0.010 (2)
C7	0.0296 (19)	0.040 (2)	0.033 (2)	0.0007 (17)	0.0126 (18)	0.0016 (18)
C19	0.0265 (18)	0.033 (2)	0.033 (2)	0.0024 (16)	0.0076 (18)	0.0031 (18)
C17	0.0296 (19)	0.0275 (18)	0.032 (2)	−0.0016 (16)	0.0032 (17)	0.0002 (17)
C6	0.033 (2)	0.030 (2)	0.054 (3)	−0.0005 (17)	0.017 (2)	0.004 (2)
C13	0.031 (2)	0.044 (2)	0.046 (3)	−0.0004 (18)	0.010 (2)	−0.009 (2)
C5	0.039 (2)	0.045 (3)	0.054 (3)	0.0063 (19)	0.024 (2)	0.018 (2)
C18	0.042 (2)	0.037 (2)	0.040 (3)	−0.0067 (18)	0.007 (2)	−0.0074 (19)
C11	0.069 (3)	0.057 (3)	0.062 (4)	0.013 (3)	0.043 (3)	0.017 (3)
C1	0.048 (3)	0.035 (2)	0.065 (3)	0.001 (2)	0.021 (2)	−0.003 (2)
C12	0.040 (2)	0.053 (3)	0.071 (4)	0.003 (2)	0.024 (3)	0.023 (3)
C14	0.045 (2)	0.057 (3)	0.033 (3)	−0.002 (2)	0.010 (2)	0.001 (2)
C10	0.064 (3)	0.055 (3)	0.040 (3)	0.004 (2)	0.028 (3)	0.006 (2)
C9	0.046 (3)	0.043 (2)	0.043 (3)	0.001 (2)	0.018 (2)	−0.001 (2)
C16	0.036 (2)	0.046 (2)	0.038 (3)	−0.0030 (19)	0.012 (2)	−0.004 (2)
C4	0.060 (3)	0.045 (3)	0.071 (4)	0.016 (2)	0.035 (3)	0.022 (3)
C2	0.069 (3)	0.038 (2)	0.092 (5)	−0.002 (2)	0.048 (3)	−0.005 (3)
C3	0.067 (3)	0.034 (2)	0.116 (6)	0.011 (2)	0.057 (4)	0.013 (3)

### Geometric parameters (Å, º)

**Table:**

Ni1—O1	2.109 (3)	C8—C9	1.383 (6)
Ni1—N3	2.133 (3)	C8—C20	1.424 (6)
Ni1—N5	2.134 (3)	C15—C14	1.361 (6)
Ni1—N2	2.159 (3)	C15—C7	1.456 (6)
Ni1—N6	2.171 (3)	C20—C12	1.396 (7)
Ni1—Cl1	2.4772 (13)	C19—C17	1.461 (6)
S1—C18	1.704 (5)	C17—C18	1.351 (6)
S1—C16	1.721 (4)	C6—C1	1.389 (7)
S2—C13	1.700 (5)	C6—C5	1.410 (6)
S2—C14	1.713 (5)	C13—H13	0.9300
O1—H1A	0.8372	C5—C4	1.387 (7)
O1—H1B	0.7947	C18—H18	0.9300
O3—N1	1.244 (5)	C11—C12	1.364 (8)
O4—N1	1.252 (5)	C11—C10	1.406 (8)
O2—N1	1.214 (5)	C11—H11	0.9300
N3—C13	1.310 (5)	C1—C2	1.381 (7)
N3—C15	1.380 (5)	C1—H1	0.9300
N5—C19	1.326 (5)	C12—H12	0.9300
N5—C8	1.386 (6)	C14—H14	0.9300
N6—C16	1.271 (6)	C10—C9	1.364 (7)
N6—C17	1.391 (5)	C10—H10	0.9300
N2—C7	1.324 (5)	C9—H9	0.9300
N2—C6	1.412 (5)	C16—H16	0.9300
N7—C19	1.354 (5)	C4—C3	1.356 (8)
N7—C20	1.367 (6)	C4—H4A	0.9300
N7—H7	0.8600	C2—C3	1.420 (8)
N4—C7	1.335 (5)	C2—H2	0.9300
N4—C5	1.379 (6)	C3—H3	0.9300
N4—H4	0.8600		
			
O1—Ni1—N3	90.37 (13)	N2—C7—C15	119.8 (4)
O1—Ni1—N5	169.10 (12)	N4—C7—C15	126.7 (4)
N3—Ni1—N5	99.19 (13)	N5—C19—N7	112.6 (4)
O1—Ni1—N2	88.88 (12)	N5—C19—C17	120.3 (3)
N3—Ni1—N2	77.42 (13)	N7—C19—C17	127.0 (4)
N5—Ni1—N2	88.12 (13)	C18—C17—N6	114.4 (4)
O1—Ni1—N6	91.69 (12)	C18—C17—C19	131.0 (4)
N3—Ni1—N6	171.22 (14)	N6—C17—C19	114.5 (3)
N5—Ni1—N6	78.08 (13)	C1—C6—C5	121.2 (4)
N2—Ni1—N6	94.09 (13)	C1—C6—N2	130.8 (4)
O1—Ni1—Cl1	91.44 (9)	C5—C6—N2	107.9 (4)
N3—Ni1—Cl1	91.99 (10)	N3—C13—S2	115.4 (4)
N5—Ni1—Cl1	93.43 (9)	N3—C13—H13	122.3
N2—Ni1—Cl1	169.41 (10)	S2—C13—H13	122.3
N6—Ni1—Cl1	96.48 (10)	N4—C5—C4	132.9 (5)
C18—S1—C16	89.2 (2)	N4—C5—C6	105.9 (4)
C13—S2—C14	89.5 (2)	C4—C5—C6	121.1 (5)
Ni1—O1—H1A	122.0	C17—C18—S1	110.3 (3)
Ni1—O1—H1B	130.8	C17—C18—H18	124.9
H1A—O1—H1B	90.7	S1—C18—H18	124.9
C13—N3—C15	110.2 (4)	C12—C11—C10	121.9 (5)
C13—N3—Ni1	134.7 (3)	C12—C11—H11	119.0
C15—N3—Ni1	115.1 (3)	C10—C11—H11	119.0
C19—N5—C8	105.7 (3)	C2—C1—C6	117.4 (5)
C19—N5—Ni1	113.7 (3)	C2—C1—H1	121.3
C8—N5—Ni1	140.4 (3)	C6—C1—H1	121.3
C16—N6—C17	111.0 (4)	C11—C12—C20	117.5 (5)
C16—N6—Ni1	135.5 (3)	C11—C12—H12	121.3
C17—N6—Ni1	113.2 (3)	C20—C12—H12	121.3
C7—N2—C6	105.0 (3)	C15—C14—S2	110.0 (4)
C7—N2—Ni1	113.3 (3)	C15—C14—H14	125.0
C6—N2—Ni1	141.4 (3)	S2—C14—H14	125.0
C19—N7—C20	107.6 (3)	C9—C10—C11	120.8 (5)
C19—N7—H7	126.2	C9—C10—H10	119.6
C20—N7—H7	126.2	C11—C10—H10	119.6
C7—N4—C5	107.6 (4)	C10—C9—C8	119.2 (5)
C7—N4—H4	126.2	C10—C9—H9	120.4
C5—N4—H4	126.2	C8—C9—H9	120.4
O2—N1—O3	120.7 (5)	N6—C16—S1	115.2 (3)
O2—N1—O4	121.6 (5)	N6—C16—H16	122.4
O3—N1—O4	117.7 (4)	S1—C16—H16	122.4
C9—C8—N5	132.1 (4)	C3—C4—C5	117.4 (5)
C9—C8—C20	119.5 (4)	C3—C4—H4A	121.3
N5—C8—C20	108.4 (4)	C5—C4—H4A	121.3
C14—C15—N3	114.9 (4)	C1—C2—C3	120.5 (5)
C14—C15—C7	130.8 (4)	C1—C2—H2	119.8
N3—C15—C7	114.3 (4)	C3—C2—H2	119.8
N7—C20—C12	133.3 (4)	C4—C3—C2	122.4 (5)
N7—C20—C8	105.7 (4)	C4—C3—H3	118.8
C12—C20—C8	121.0 (5)	C2—C3—H3	118.8
N2—C7—N4	113.5 (4)		

### Hydrogen-bond geometry (Å, º)

**Table:**

*D*—H···*A*	*D*—H	H···*A*	*D*···*A*	*D*—H···*A*
O1—H1*A*···O3^i^	0.84	2.00	2.751 (5)	149
O1—H1*B*···O2^ii^	0.79	2.53	3.293 (6)	160
O1—H1*B*···O4^ii^	0.79	2.38	3.025 (5)	139
N4—H4···O3^iii^	0.86	2.35	2.962 (5)	129
N4—H4···O4^iii^	0.86	2.14	2.996 (5)	173
N7—H7···Cl1^iv^	0.86	2.35	3.159 (4)	157

Symmetry codes: (i) *x*−1, *y*, *z*; (ii) −*x*+1, *y*−1/2, −*z*+1/2; (iii) *x*−1, −*y*+3/2, *z*−1/2; (iv) −*x*+1, −*y*+1, −*z*+1.
